# Gray matter abnormalities in patients with major depressive disorder and social anxiety disorder: a voxel-based meta-analysis

**DOI:** 10.1007/s11682-023-00797-z

**Published:** 2023-09-19

**Authors:** Junquan Liang, Qiaoyun Yu, Yuchen Liu, Yidan Qiu, Rundong Tang, Luda Yan, Peng Zhou

**Affiliations:** 1 Shenzhen Bao’an Chinese Medicine Hospital, The Seventh Clinical Medical School of Guangzhou University of Chinese Medicine, Shenzhen, 518101 Guangdong China; 2 The Brain Cognition and Brain Disease Institute (BCBDI), Shenzhen Institute of Advanced Technology, Chinese Academy of Sciences (CAS), Shenzhen-Hong Kong Institute of Brain Science-Shenzhen Fundamental Research Institutions, Shenzhen, Guangdong China; 3 University of Chinese Academy of Sciences, Beijing, China; 4 Jingzhou Traditional Chinese Medicine Hospital, Jingzhou, Hubei China; 5 Shenzhen Luohu District Hospital of TCM, Shenzhen, Guangdong China; 6 Centre for the Study of Applied Psychology, Guangdong Key Laboratory of Mental Health and Cognitive Science, School of Psychology, Institute for Brain Research and Rehabilitation, South China Normal University, Guangzhou, Guangdong China

**Keywords:** Major depressive disorder, Social anxiety disorder, Meta-analysis, Gray matter, Voxel-based morphometry

## Abstract

**Background:**

Major depressive and social anxiety disorders have a high comorbidity rate and similar cognitive patterns. However, their unique and shared neuroanatomical characteristics have not been fully identified.

**Methods:**

Voxel-based morphometric studies comparing gray matter volume between patients with major depressive disorder/social anxiety disorder and healthy controls were searched using 4 electronic databases from the inception to March 2022. Stereotactic data were extracted and subsequently tested for convergence and differences using activation likelihood estimation. In addition, based on the result of the meta-analysis, behavioral analysis was performed to assess the functional roles of the regions affected by major depressive disorder and/or social anxiety disorder.

**Results:**

In total, 34 studies on major depressive disorder with 2873 participants, and 10 studies on social anxiety disorder with 1004 subjects were included. Gray matter volume conjunction analysis showed that the right parahippocampal gyrus region, especially the amygdala, was smaller in patients compared to healthy controls. The contrast analysis of major depressive disorder and social anxiety disorder revealed lower gray matter volume in the right lentiform nucleus and medial frontal gyrus in social anxiety disorder and lower gray matter volume in the left parahippocampal gyrus in major depressive disorder. Behavioral analysis showed that regions with lower gray matter volume in social anxiety disorder are strongly associated with negative emotional processes.

**Conclusions:**

The shared and unique patterns of gray matter volume abnormalities in patients with major depressive and social anxiety disorder may be linked to the underlying neuropathogenesis of these mental illnesses and provide potential biomarkers.

**PROSPERO registration number:** CRD42021277546.

**Supplementary Information:**

The online version contains supplementary material available at 10.1007/s11682-023-00797-z.

## Introduction

From the perspective of etymology, the word *comorbidity* first appeared in 1985, composed of *co-* "along with" and *morbidity* "diseased condition." The co-existence of MDD and SAD is one of the most common comorbidities among mental illnesses, and the incidence ranges from 19.5% to 74.5% (Arditte Hall et al., 2019; Ohayon & Schatzberg, 2010; Zhao et al., 2017). According to the fifth edition of the DSM, MDD manifests as a persistent low mood and/or a loss of interest or pleasure in usual activities (Trivedi, 2020), while an intense fear of social situations is characteristic of SAD (Leichsenring & Leweke, 2017). Although MDD and SAD both are disabling psychiatric disorders, comorbidity can lead to more severe outcomes (Adams et al., 2016). Currently, it is assumed that MDD and SAD may have a similar etiology and pathophysiological basis and neuroanatomical characteristics (Zhao et al., 2020).

With the continuous development of neuroimaging techniques, studies have focused on the unique and shared neuroanatomical characteristics of MDD and SAD. VBM is a neuroimaging technique that investigates focal differences in brain anatomy by segmenting the brain into gray matter, white matter, and cerebrospinal fluid and warping the segmented images to template space (Nemoto, 2017). Based on this technique, clinical studies have shown decreased GM volumes of frontal and temporal regions in patients with MDD compared with HCs (Kandilarova et al., 2019). Other studies suggested that the anterior insula GM is strongly affected in MDD and may play an important role in the neuropathogenesis of depression (Stratmann et al., 2014). Recently, researchers found that patients with MDD have decreased GM volume in various regions, including the superior temporal cortex, anterior and middle cingulate cortex, inferior frontal cortex, and precuneus (Wang et al., 2022). A meta-analysis of VBM studies reported abnormalities of the subgenual cingulate cortex, hippocampus, amygdala, and putamen in patients with MDD (Gray et al., 2020). A multi-center mega-analysis of SAD by VBM claimed that patients with SAD had larger GM volumes in the dorsal striatum compared with HCs (Bas-Hoogendam et al., 2017). In the meta-analysis, patients with SAD had larger GM in the left precuneus, right MOG, and SMA but smaller GM in the left putamen (Wang et al., 2018). However, there are several issues regarding these results. First, the small and heterogeneous sample sizes led to controversial results from VBM studies, a common problem that needs to be solved in neuroimaging studies. Second, even with the increasing number of studies on GM differences in MDD or SAD, the results remained inconsistent. For example, some studies have reported decreased GM volume in the prefrontal cortex, the amygdala, or the hippocampus of patients with MDD or SAD compared to HCs, while others have found no significant differences or even increased GM volume in these regions. Last but not least, the cognition patterns of the two disorders are similar, but their symptoms are different and unique or shared neuroanatomical characteristics have not been fully identified. Undoubtedly, controversial and inconsistent results have limited our understanding of the exact neuropathogenesis of MDD and SAD. The continuous development of meta-analysis methods in neuroimaging may solve this problem, as it is now possible to pool data from relevant studies to identify brain regions that are associated with the disease. The ALE approach uses the probability distribution model to determine the consistency of activated brain regions across several studies (Eickhoff et al., 2009). Many studies indicated that the ALE approach can be applied to identify the neuroanatomical characteristics of diseases, especially for complex comorbidities of psychiatric disorders. However, to date, there are still no voxel-based meta-analysis studies based on ALE approach to compare GM abnormalities in MDD and SAD.

Therefore, we performed a voxel-based meta-analysis of VBM studies using the ALE method and GingerALE software to investigate the unique and shared GM characteristics of patients with MDD and SAD compared with HC participants. Our findings can help understand the neuroanatomical alterations related to the comorbidities of psychiatric disorders and optimize the diagnosis and treatment of mental illnesses.

## Materials and methods

### Study registration

Our study has been registered in PROSPERO (registration number: CRD42021277546). The review reporting was conducted in compliance with the Preferred Reporting Items for Systematic Reviews and Meta-analyses (PRISMA) guidelines.

### Literature search and included/excluded criteria

We searched four international electronic databases (PubMed, EMBASE, ScienceDirect, and Web of Science) from the inception to March 2022 to identify relevant studies. The search terms were major depressive disorder, social anxiety disorder, and VBM and were adapted for each database as necessary. The references of the included studies were also screened to find further studies. The detailed search strategy for PubMed is in Supplemental Table 1. The same search strategy was used for other electronic databases.

Studies were included if they met the following inclusion criteria: (1) patients met the diagnostic criteria for MDD or SAD; (2) Studies containing coordinate-based results in a standard reference space (MNI/Talairach); (3) Original peer-reviewed articles published in English; (4) For studies with intervention, only baseline data were included. Studies with the following characteristics were excluded: (1) Review articles, research protocols, letters, commentaries, or meta-analyses; (2) Studies reporting only ROI findings or using seed voxel-based analysis method; (3) Studies with a sample size of less than ten; (4) Lack of peak coordinates of significant clusters. Authors of published reports were contacted by email when the required information was not provided.

### Study selection and data extraction

As the first step in data processing, titles and abstracts of all studies were screened for relevance, and irrelevant were excluded.

In the second step, two members of the review team (Qiaoyun Yu and Yuchen Liu) independently assessed the eligibility of the studies using the predefined inclusion and exclusion criteria. Besides, for the studies that met the inclusion criteria, the whole article was studied by reviewers to ensure that the entire study met the criteria and was prepared to extract relevant information. Disagreements on including a specific study were resolved by discussion between the reviewers. The missing information was collected by contacting the authors of the original article.

The data that were extracted by the review team included: the study setting, study population, participant demographics, coordinates associated with GM volumes, study methodology, multiple comparison corrections, MRI scanner, and smoothing kernel. Besides, peak coordinates with statistically significant differences at the whole-brain level (no small volume correction, SVC) were extracted.

### Quality assessment of MRI studies

To ensure the reliability of neuroimaging data, we adopted criteria for the quality of MRI reporting that dictate a more consistent and coherent policy for reporting MRI (Poldrack et al., 2008).

### Data synthesis

Statistically significant differences between MDD/SAD and HCs groups were extracted and recorded for each study. Lancaster transforms (icbm2tal) incorporated in GingerALE was used to convert coordinates from Talairach coordinates to MNI space if necessary. All meta-analyses were performed using GingerALE (https://www.brainmap.org/ale). The coordinates were extracted from included studies and weighted according to the sample size (number of participants). These weightings contributed to forming estimates of ALE for each intracerebral voxel on a standardized map.

Some planned analyses were conducted. (1) ALE analyses were performed separately to compare MDD vs. HCs and SAD vs. HCs, with an initial threshold of voxel-level *P* < 0.05 and a minimum cluster size of 20 mm^3^. (2) The results of ALE were used for a subsequent conjunction/contrast analysis to measure the unique and shared neuroanatomical alterations of MDD and SAD. Quantitative conjunction analysis and non-parametric permutation simulations (10,000 permutations) were applied to determine the statistical inferences of differences between subjects with MDD and subjects with SAD. The statistical significance of the ALE results was determined by a permutation test, setting cluster-level inference at *P* < 0.05 (False discovery rate, FDR). The results (ALE maps) were visualized using Mango (http://ric.uthscsa.edu/mango) overlaid onto a standardized anatomical template (the ICBM-152 brain template) (Lancaster et al., 2007).

The primary outcome was morphological brain differences measured by VBM between HCs and patients with MDD comorbid with SAD and pooling all results to examine the neuroanatomical alterations associated with these psychiatric disorders. Planned subgroup analyses were as follows: The first subgroup analysis only included results that had been corrected for multiple comparisons in the original study. Next, we ran two subgroup analyses with the difference of datasets acquiring images with a 3.0 T or 1.5 T MRI scanner or the datasets provided by a smoothing kernel of 8 mm, 10 mm, or 12 mm.

The behavior analysis plugin of Mango software was used to perform regional behavior analysis based on the selected brain ROI. Since the analysis was coordinate-based, it could verify whether the origin was correctly positioned (press the 'o' key in Mango). The method of conducting behavioral analysis using the Mango software is as follows: First, we determined the ROI based on the research results. Second, we ran the behavior analysis plugin on the selected ROI. The plugin compares the brain activity in the ROI with the BrainMap database, which contains information on the behavioral domains and sub-domains associated with different brain regions. The plugin then presents the analysis results for the five behavioral domains (Action, Cognition, Emotion, Interoception, and Perception) and sixty sub-domains. The plugin also provides a behavior profile chart and a *Z*-score ranked table to help interpret the results. Third, we interpreted and reported the results of the behavior analysis. Only *Z*-scores ≥ 3.0 were considered statistically significant (*P* < 0.05 with Bonferroni correction for multiple comparisons).

## Results

### Study description and participants

**Fig. 1:**
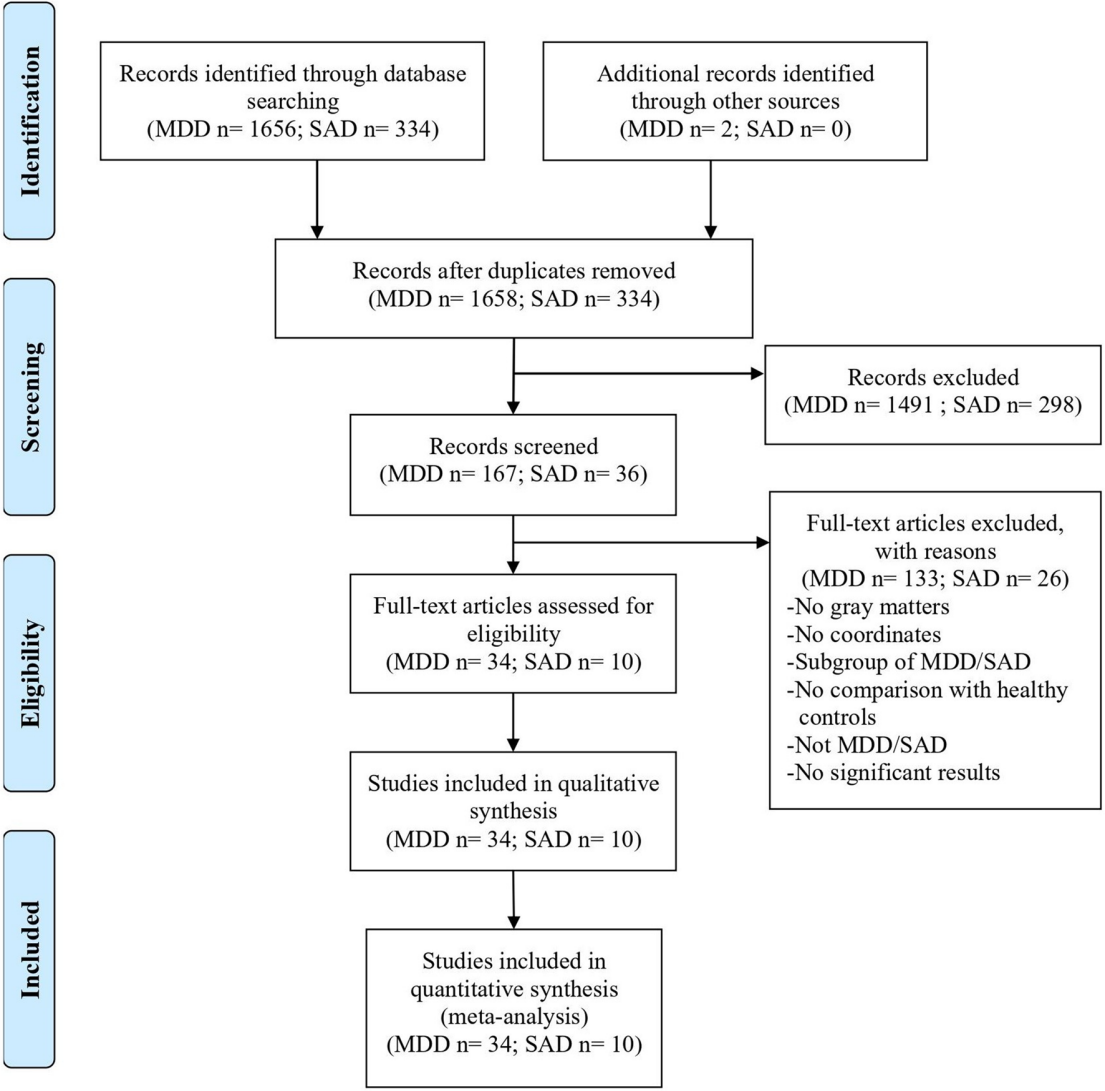
Flow diagram of the study selection process

We obtained 1992 relevant studies (MDD: 1658; SAD: 334) through preliminary searches. After multiple filtering steps, 42 articles with 3723 participants were included, including 34 studies on MDD and 10 studies on SAD. The flowchart of the study selection procedure is shown in Fig. 1.

The 34 included studies on MDD involved 2873 participants, consisting of 1512 patients with MDD and 1361 HCs. Ten studies on SAD contained 464 patients with SAD and 540 HCs. Among these studies, almost all of them reported basic information such as sample size, age, and gender. There were 14 studies on MDD and 8 studies on SAD that described the duration of the disease. Regarding handedness, 25 studies on MDD and 6 studies on SAD reported a dominant hand. The detailed characteristics of the included studies are shown in Table 1.

**Table 1:** Characteristics of the included studies

NO	Included studies (author, year)	Age (years)		Sample size (MDD or SAD group/HC group)	Sex (F/M)		Handedness (right/left)		Age of onset (years)	Illness duration (years/ months/ weeks/ days)	MRI scanner (T)	Smoothing kernel (mm)
		MDD/SAD group	HC group		MDD/SAD group	HC group	MDD/SAD group	HC group				
1. MDD (*n* = 34)												
1	Alemany et al., 2013	/	34.9 ± 8.0	18/21	/	9/12	10/0	10/0	/	/	3	12
2	Arnone et al., 2013	Currently MDD: 36.3 ± 8.8; Remitted MDD: 34.5 ± 11.0	32.1 ± 9.3	64/66	Currently MDD: 27/12; Remitted MDD: 20/5	46/20	64/0	64/0	Currently MDD: 22.0 ± 8.1; Remitted MDD: 25.1 ± 10.8	Currently MDD: 21.4 ± 18.9 (weeks)	1.5	8
3	Bergouignan et al., 2009	33.16 ± 9.58	28.21 ± 5.50	21/21	17/4	14/7	21/0	21/0	23.8 ± 8.65	4.56 ± 3.22 (months)	1.5	/
4	Chaney et al., 2014	MDD without maltreatment: 40.6 ± 10.4; MDD with maltreatment: 39.9 ± 9.7	HC without maltreatment: 34.2 ± 10.8; HC with maltreatment: 45.3 ± 15.8	37/46	MDD without maltreatment: 12/5; MDD with maltreatment: 9/11	MDD without maltreatment: 24/12; MDD with maltreatment: 4/6	/	/	MDD without maltreatment: 26.3 ± 10.9; MDD with maltreatment: 22.0 ± 12.1	MDD without maltreatment: 7.6 ± 8.5; MDD with maltreatment: 11.4 ± 11.3 (years)	3	10
5	Frodl et al., 2008	46.1 ± 11.3	43.6 ± 11.3	38/30	25/13	19/11	34/4	28/2	40.0 ± 12.1	24.9 ± 21.0 (months)	1.5	8
6	Grieve et al., 2013	33.8 ± 13.1	31.5 ± 12.4	102/34	54/48	16/18	/	/	22.1 ± 12.2	11.3 ± 11.8 (years)	3	8
7	Hwang et al., 2010	79.4 ± 5.3	79.5 ± 4.3	70/26	0/70	0/26	/	/	68.8 ± 11.4	6.5 ± 5.8 (years)	2	8
8	Kim et al., 2008	38.5 ± 9.70	35.3 ± 11.25	22/25	22/0	25/0	19/3	21/4	17.4 ± 10.01	6.5 ± 3.33 (years)	1.5	12
9	Lai et al., 2015	40.07 ± 8.99	40.38 ± 10.51	53/54	28/25	28/25	52/1	52/2	/	5.03 ± 1.62 (months)	3	3
10	Machino et al., 2014	39.57 ± 8.29	38.66 ± 8.36	29/29	13/16	13/16	/	/	34.72 ± 7.56	52.55 ± 57.81 (months)	1.5	8
11	Mak et al., 2009	45.5 ± 8.5	45.8 ± 9.8	17/17	17/0	17/0	17/0	17/0	/	/	1.5	12
12	Peng et al., 2011	46.7 ± 8.9	45.9 ± 9.0	22/30	14/8	19/11	22/0	30/0	/	8.6 ± 6.5 (months)	3	12
13	de Azevedo-Marques Périco et al., 2011	29.9 ± 8.9	30.2 ± 8.4	20/94	15/5	41/53	17/3	91/3	/	Affective symptoms: 245.9 ± 201.4; Psychotic symptoms: 41.1 ± 47.8 (days)	1.5	8
14	Scheuerecker et al., 2010	37.9 ± 10.1	35.5 ± 10.9	13/15	3/10	5/10	13/0	15/0	/	52.3 ± 71.5 (months)	3	8
15	Shah et al., 1998	48.9 ± 9.8	49.3 ± 11.8	20/20	7/13	7/13	/	/	38.9 ± 13.5	197 ± 125 (weeks)	1	12
16	Stratmann et al., 2014	37.86 ± 11.87	37.82 ± 11.42	132/132	76/56	74/58	132/0	132/0	34.86 ± 11.69	/	3	8
17	Tang et al., 2007	29.5 ± 6.84	29.46 ± 6.86	14/13	14/0	13/0	/	/	/	5.44 ± 5.22 (months)	1.5	8
18	van Tol et al., 2010	37.16 ± 10.24	40.54 ± 9.71	68/65	44/24	41/24	62/6	60/5	25.62 ± 10.36	/	3	8
19	Vasic et al., 2008	37.4 ± 8.5	31.4 ± 9.6	15/14	6/9	6/8	15/0	14/1	/	43.4 ± 37.3 (months)	1.5	8
20	Wagner et al., 2011	High suicide risk MDD patients: 41.0 ± 12.5; Non-high risk MDD patients: 34.1 ± 10.5	35.1 ± 10.4	30/30	High suicide risk MDD patients: 11/4; Non-high risk MDD patients: 14/1	25/5	30/0	30/0	High suicide risk MDD patients: 32.1 ± 12.7; Non-high risk MDD patients: 31.1 ± 10.3	High suicide risk MDD patients: 8.9 ± 9.4; Non-high risk MDD patients: 3 ± 3.2 (years)	1.5	12
21	Zhang et al., 2012	20.52 ± 1.72	21.03 ± 1.47	33/32	16/17	15/17	33/0	32/0	/	/	1.5	8
22	Zou et al., 2010	31.1 ± 10.4	36.6 ± 12.9	23/23	13/10	13/10	23/0	23/0	/	7.6 ± 4.4 (months)	3	/
23	Straub et al., 2019	17.30 ± 3.44	17.62 ± 3.85	60/43	48/12	38/5	58/2	40/3	/	/	3	6
24	Wang et al., 2019	MDD with a history of suicide attempts: 27.61 ± 10.536; MDD without a history of suicide attempts: 28.13 ± 7.617	25.83 ± 5.898	98/60	MDD with a history of suicide attempts: 27/11; MDD without a history of suicide attempts: 47/13	43/17	/	/	/	MDD with a history of suicide attempts: 32.63 ± 83.17; MDD without a history of suicide attempts: 17.17 ± 29.08 (months)	3	8
25	Li et al., 2019	35.1 ± 8.9	30.7 ± 8.068/65	56/56	36/20	23/33	56/0	56/0	/	1.1 ± 1.3 (years)	3	8
26	Sun et al., 2020	36.30 ± 11.015	33.61 ± 8.086	30/30	15/15	11/20	30/0	30/0	/	/	3	6
27	Chen et al., 2020	28.7 ± 13.3	27.4 ± 10.2	22/22	18/4	18/4	/	/	24.3 ± 13.1	0.2 ± 0.2 (years)	3	8
28	Zhang et al., 2020a (1)	MDD with suicidal ideation: 31.03 ± 10.21; MDD without suicidal ideation: 28.79 ± 9.56	29.63 ± 8.90	73/43	MDD with suicidal ideation: 22/13; MDD without suicidal ideation: 27/11	27/16	64/9	39/4	/	MDD with suicidal ideation: 26.91 ± 47.25; MDD without suicidal ideation: 27.91 ± 44.67 (months)	3	8
29	Xu et al., 2019	39.27 ± 7.84	/	11/12	6/5	/	11/0	12/0	/	25.82 ± 3.63 (months)	3	8
30	Meng et al., 2020	33.71 ± 10.21	35.64 ± 8.668	159/53	83/76	25/27	159/0	53/0	/	/	3	8
31	Lu et al., 2019	MDD patients with childhood trauma exposures: 24.4 ± 4.79; MDD patients without childhood trauma exposures: 23.5 ± 5.77	HC with childhood trauma exposures: 21.5 ± 3.98; HC without childhood trauma exposures: 21.5 ± 3.69	30/48	HC with childhood trauma exposures: 5/11; HC without childhood trauma exposures: 8/6	HC with childhood trauma exposures: 15/9; HC without childhood trauma exposures: 15/9	30/0	48/0	/	HC with childhood trauma exposures: 39.1 ± 32.4; HC without childhood trauma exposures: 21.1 ± 19.2 (months)	3	8
32	Ma et al., 2021	24.98 ± 4.8	25.25 ± 4.07	52/65	34/18	32/33	52/0	65/0	/	/	3	8
33	Burhanoglu et al., 2021	23.22 ± 1.91	22.34 ± 1.86	30/29	30/0	29/0	/	/	15.73 ± 2.65	/	3	8
34	Zhang et al., 2020b (2)	25.0 (21.8–48.3)	23.0 (22.0–29.0)	30/63	21/9	39/24	30/0	63/0	/	/	3	10
2. SAD (*n* = 10)												
1	Talati et al., 2013	31.5 ± 9.1	31.5 ± 9.1	33/37	24/9	19/18	/	/	11.0 ± 5.9	/	1.5	8
2	Bas-Hoogendam et al., 2017	30.6 ± 10.0	32.4 ± 10.5	174/213	102/72	116/9	172/2	206/7	14.8 ± 7.1	/	3	3
3	Irle et al., 2014	31.0 ± 10.0	32.0 ± 10	67/64	35/32	31/33	62/5	56/6	16.0 ± 6.0	15.0 ± 9.0 (years)	3	8
4	Kawaguchi et al., 2016	36.2 ± 11.8	33.8 ± 9.6	13/18	8/5	12/6	/	/	13.0 ± 10.3	23.3 ± 14.4 (years)	3	8
5	Meng et al., 2013	21.80 ± 3.68	21.58 ± 3.72	20/19	6/14	6/13	/	/	Below 18 years: 13.31 ± 3.11 After 18 years: 20.94 ± 4.71	50.50 ± 45.82 (months)	3	12
6	Tükel et al., 2015	27.70 ± 6.67	27.70 ± 5.83	27/27	15/12	15/12	27/0	27/0	14.52 ± 4.10	13.76 ± 6.99 (years)	1.5	8
7	Zhao et al., 2017	26.7 ± 7.1	27.1 ± 7.2	24/41	9/15	15/26	24/0	41/0	/	7.6 ± 3.8 (years)	3	10
8	Liu et al., 2021	16.28 ± 0.76	16.49 ± 0.34	31/42	21/10	23/19	/	/	/	27.12 ± 28.20 (months)	3	8
9	Mansson et al., 2016	32.3 ± 9.7	32.2 ± 10.5	26/26	22/26	18/26	26/0	26/0	15.9 ± 6.0	/	3	8
10	Zhang et al., 2022	24.6 ± 5.3	23.4 ± 3.3	49/53	30/19	31/22	49/0	53/0	/	7.2 ± 4.1	3	6

Zhang et al., 2020a (1): the first author was Zhang Ran; Zhang et al., 2020b (2): the first author was Zhang Yiran

### Quality of MRI studies

We assessed the quality of included studies using guidelines for the standardized reporting of MRI studies. All 44 studies reported MRI design, ethics approval, software package, image acquisition, processing, and analysis. Besides, 37 articles described multiple comparison corrections, accounting for 84% of all included studies (Supplemental Table 2). Collectively, the quality of the MRI studies was moderate.

### Voxel-based meta-analysis

#### Conjunction and contrast voxel-based meta-analysis in MDD and SAD

**Fig. 2:**
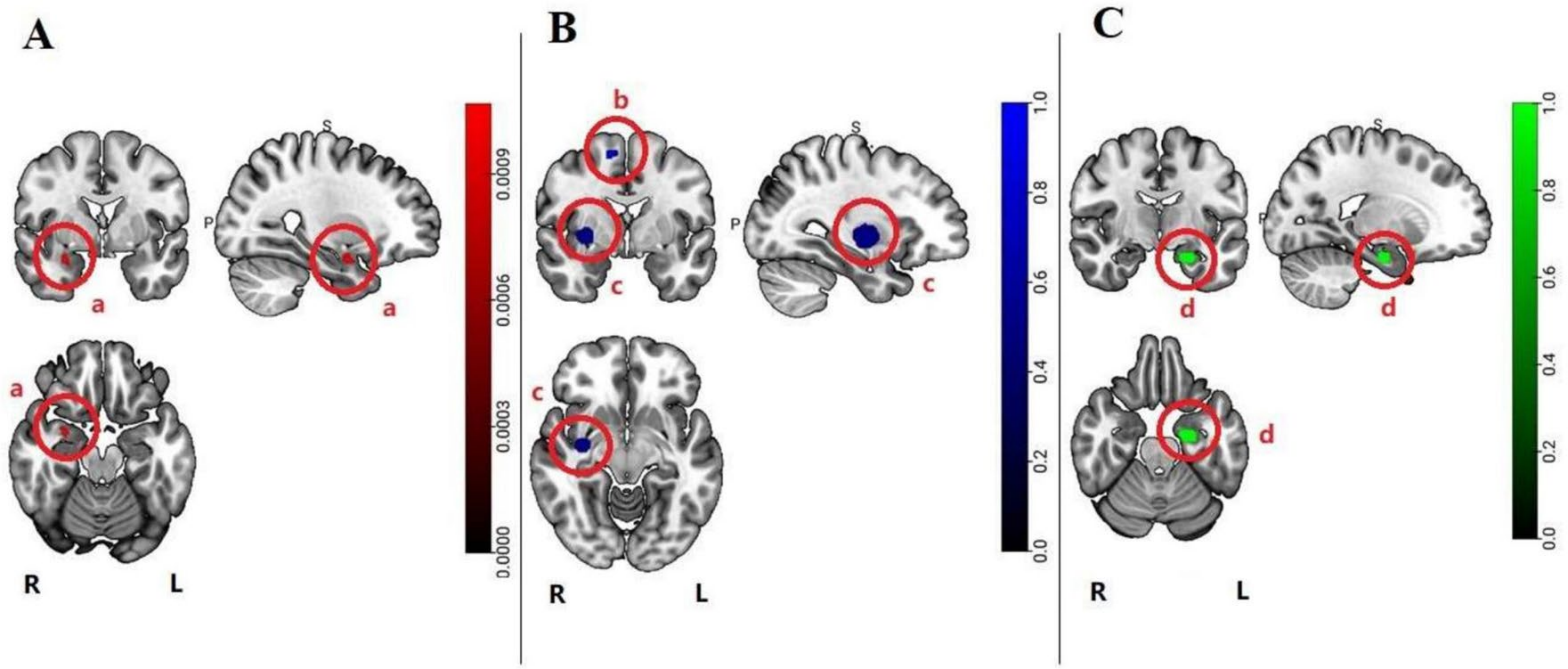
The pattern of GM abnormalities in MDD and SAD is based on a voxel-based meta-analysis. (Radiology view. permutation test *P* < 0.05, FDR corrected) **(A)** Pattern of overlapping GM volume decrease between MDD and SAD, **a**: Right parahippocampal gyrus; **(B)** GM volumes lower in SAD, **b**: Right lentiform nucleus, **c**: Right medial frontal gyrus; **(C)** GM volumes lower in MDD, **d**: Left parahippocampal gyrus

Compared with HCs, conjunction analysis showed smaller volumes of GM in the parahippocampal gyrus, particularly in the amygdala, of patients with MDD and SAD (Fig. 2A, Table 2A). Contrast voxel-based meta-analysis revealed lower GM volume in the right lentiform nucleus and right medial frontal gyrus in SAD compared to MDD (Fig. 2B, Table 2B). In addition, GM volume was lower in the left parahippocampal gyrus in MDD compared to SAD (Fig. 2C, Table 2C).

**Table 2:** The clusters characteristics of GM volumes abnormalities overlapping and distinct patterns in MDD and SAD

Cluster	Volume (mm^3^)	Region	Peak coordinates			Hemisphere
			X	Y	Z	
A. Overlapping decrease in MDD and SAD						
1	33	Parahippocampal Gyrus (Amygdala)	30	0	-22	R
B. GM volumes greater decrease in SAD						
1	537	Lentiform Nucleus	34	-10	2	R
2	60	Medial Frontal Gyrus	10	-2	62	R
C. GM volumes greater decrease in MDD						
1	126	Parahippocampal Gyrus	-26	-12	-26	L
D. GM volumes greater decrease in SAD (All studies corrected for multiple comparisons)						
1	11,008	Lentiform Nucleus	36.5	-10	-8	R

#### Subgroup analysis voxel-based meta-analysis in MDD and SAD

1. Subgroup analysis of studies corrected for multiple comparisons
  Nine articles on MDD (Chen et al., 2020; Hwang et al., 2010; Mak et al., 2009; Peng et al., 2011; Scheuerecker et al., 2010; Shah et al., 1998; Tang et al., 2007; Vasic et al., 2008; Zou et al., 2010) and 2 articles on SAD (Irle et al., 2014; Talati et al., 2013) that were not corrected for multiple comparisons, were removed from contrast. After correcting for multiple comparisons, the ALE showed that GM volume decrease was greater in SAD than in MDD. Despite having a greater volume decrease, this cluster was largely identical to the cluster identified in primary outcomes (in the right lentiform nucleus), (Table 2D).
2. Subgroup analysis of studies acquired images with a 3.0 T or 1.5 T MRI scanner.
  Twenty-one studies on MDD and 8 studies on SAD used a 3 T MRI scanner, and 11 studies on MDD and 2 studies on SAD used a 1.5 T MRI scanner in this subgroup analysis. The ALE analysis revealed no significant differences at the voxel-level *P*< 0.05, cluster level FDR corrected *P*< 0.05 threshold, and a minimum cluster size of 20 mm^3^.
3. Subgroup analysis of studies using a smoothing kernel of 8 mm, 10 mm, or 12 mm.
  In total, 21 studies on MDD and 7 studies on SAD used an 8 mm smoothing kernel; 2 studies on MDD and 1 study on SAD used a 10 mm smoothing kernel; and 6 studies on MDD and 1 study on SAD used a 12 mm smoothing kernel. The subgroup analysis showed no significant differences at the voxel-level *P*< 0.05, cluster level FDR corrected *P*< 0.05 threshold, and a minimum cluster size of 20 mm^3^.

#### Regional behavior analysis

**Fig. 3:**
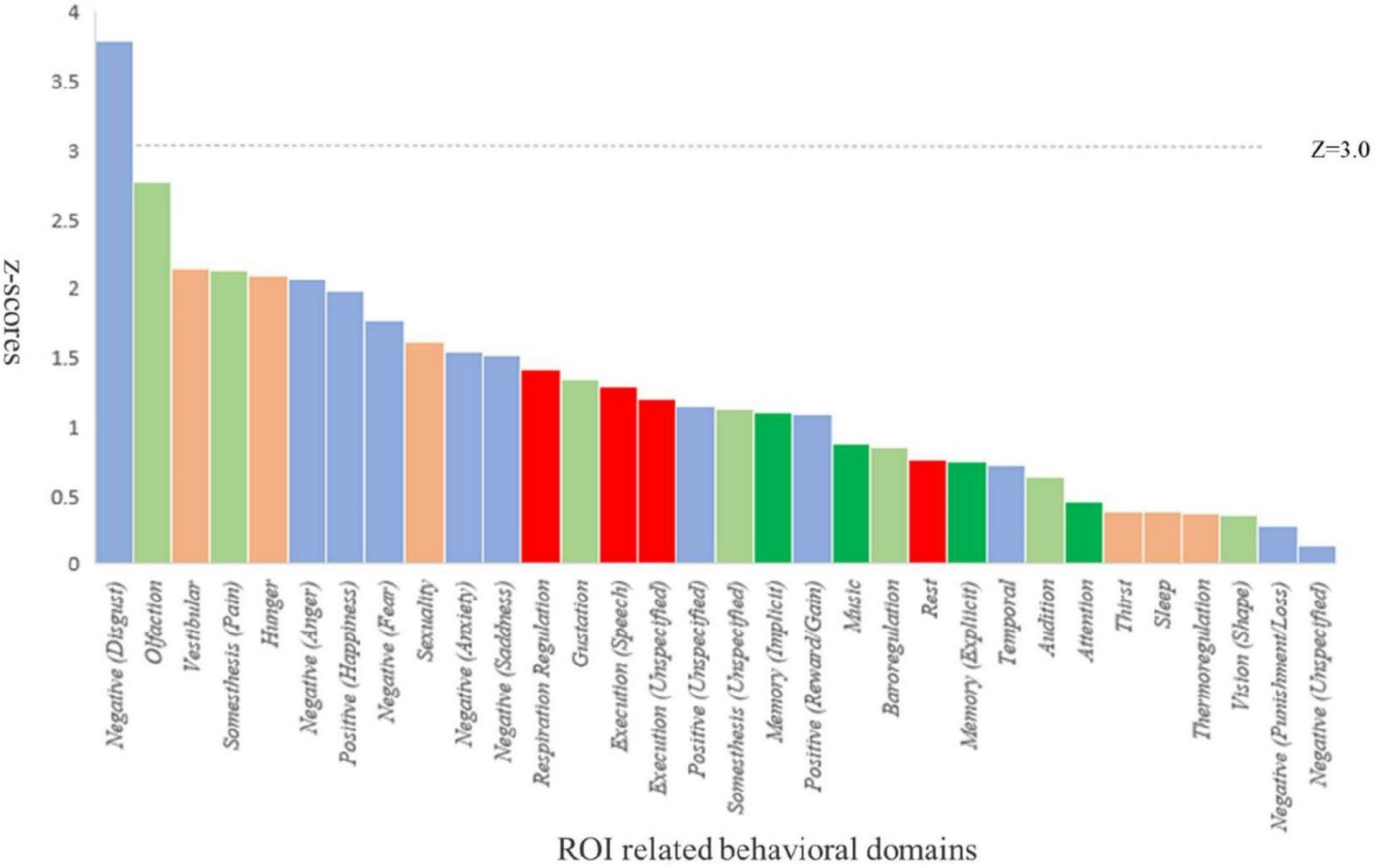
The behavioral analysis results of the regions showing lower GM volume in SAD

Behavioral analysis helps understand the relationship between brain function and behavior. By analyzing brain activity in different regions and comparing them with the behavioral domains and sub-domains, we can uncover the neural mechanisms underlying various cognitive, emotional, and perceptual processes. Therefore, using the BrainMap database, we performed a behavioral analysis based on the results of conjunction and contrast analysis to assess the functional roles of regions with abnormal GM volumes. The behavioral analysis demonstrated that regions with lower GM volumes in SAD were strongly associated with negative emotional processes (*Z* ≥ 3, *P* < 0.05, Bonferroni corrected) (Fig. 3).

## Discussion

### Summary of main results

We included 34 studies on MDD and 10 studies on SAD comprising 1512 patients with MDD and 464 patients with SAD. We performed a voxel-based meta-analysis of these studies to compare alterations in GM volume in patients with MDD and SAD and identify their unique and shared neuroanatomical characteristics. The meta-analysis revealed that:

The GM abnormalities in the right parahippocampal gyrus, in the amygdala, may be related to similar cognitive patterns and high comorbidity rate of MDD and SAD. Patients with MDD or SAD have similar ruminations and concerns, as a negative cognition pattern, and subjects with both MDD and SAD have more severe symptoms (Arditte Hall et al., 2019). Although MDD and SAD seem to be two unrelated psychiatric disorders, the high comorbidity rate and similar cognitive patterns indicate a common neuropathogenesis. From the perspective of histopathological studies, patients with MDD have reduced synaptic markers and glial cells in the parahippocampal gyrus, prefrontal cortex, amygdala, hippocampus, and raphe nucleus (Drevets, 2004). Normal neural structure in the parahippocampal gyrus and amygdala plays a significant role in affective function and memory (Baxter & Murray, 2002). Compelling evidence suggests that MDD is associated with glutamatergic transmission dysfunction in the brain and that depression may disrupt glutamate signaling through the glutamatergic NMDA receptor in the amygdala (Karolewicz et al., 2009). The amygdala is also a critical center of neuronal plasticity for fear conditioning (Klumpp & Fitzgerald, 2018). Intense fear of social situations is the typical characteristic of SAD. Recent studies suggested that genetic and environmental factors explain most individual differences among patients with SAD. Neurobiological studies have shown that dysfunctional neuronal circuits of the amygdala, insula, hippocampus, and orbital frontal regions and serotonin dysregulation are involved in SAD (Leichsenring & Leweke, 2017). We found that MDD and SAD are both linked to the dysfunction of the amygdala. Our results are consistent with previous findings and support the concept of shared pathological processes in MDD and SAD. Consistent with previous studies, we found that only the right amygdala was associated with lower GM volume, which may be related to different substructures of contralateral amygdalas (Jung et al., 2018; Roddy et al., 2021).

Lower GM volume in the right lentiform nucleus and medial frontal gyrus in SAD may be its unique neuro-structural characteristic compared to MDD. The lentiform nucleus plays a key role in the basal ganglia circuitry, composed of the pallidum and putamen. Based on neurofunctional and neuroanatomic studies of pediatric patients with bipolar disorder, researchers have confirmed that lentiform nuclei is responsible for diverse functions, including information transfer to the prefrontal cortex, reward processing, and visuospatial processing (Strawn et al., 2016). Further studies unfolded that patients with fear-based anxiety disorders exhibit disorder-specific connectivity in their thalamic nuclei, including lentiform (Etkin et al., 2009). Recent neuroimaging studies revealed that the functions of the lentiform nucleus are related to working memory and processing speed. They also indicated that there are functional differences between contralateral lentiform nuclei (Li et al., 2021). It is well known that the caudate nucleus and lenticular nucleus constitute the striatum. The neural function network between the lenticular nucleus and the frontal cortex is engaged in decision-making during adaptive goal-directed behaviors (Friedman et al., 2015). Electrophysiological studies illuminated that the neural trajectories in the medial frontal cortex and striatum show increased spike synchrony during processing decision-related information (Handa et al., 2021). Accumulating evidence reveals that there is a close link between avoidant decisions and anxiety in patients with SAD. Individuals who showed a deficit in the goal-directed adjustment of their decisions had higher and sustained distress in response to social stressors and reported a slightly decreased avoidance following treatment (Pittig et al., 2015). These findings point out a critical phenomenon, named avoidant decisions, which is closely related to the development and prognosis of SAD. The functional assembly of the middle frontal gyrus-striatum (lentiform nucleus) plays an important role in avoidant decisions. Additionally, sequential studies demonstrated that the interaction of the frontal cortex with the striatum is critical for generating and regulating negative emotion (Hiser & Koenigs, 2018). This is consistent with the results of our behavioral analysis, showing the role of the lenticular nucleus and middle frontal gyrus in regulating negative emotions. Further subgroup analysis based on multiple comparison corrections proved the stability of these results. Negative emotional processes are "unpleasant and disruptive emotional reactions" that interfere with our normal functioning and goals. They are associated with experiencing and expressing negative emotions, such as anger, anxiety, fear, apathy, contempt, hate, disgust, jealousy, insecurity, regret, guilt, sadness, grief, loneliness, and shame. Negative emotions also have adverse effects on decision-making function (Tao et al., 2023), and may be closely related to SAD. Our results highlighted the middle frontal gyrus-striatum (lentiform nucleus) functional assembly as the unique neuroanatomical characteristics of SAD, which discriminates it from MDD. Therefore, simultaneous abnormalities of the lentiform nucleus and medial frontal gyrus may be a better neuroimaging marker for SAD.

Compared with SAD, GM volume decrease in the left parahippocampal gyrus is greater in MDD. Unlike the extensive pattern of GM volume decrease in patients with SAD, patients with MDD present with a different pattern of GM abnormalities. Compared with SAD, the GM volume decrease in the left parahippocampal gyrus is greater in MDD. The important role of the parahippocampal gyrus in the pathogenesis of MDD has been discussed in previous studies (Milne et al., 2012). This section focuses on the mechanism behind the unique abnormal neuroanatomical structures of MDD compared with SAD. Our analysis of behavioral domain profiles indicated that the affected region in MDD was related to negative emotion processing and mirrored the clinical deficits. According to the cognitive theories of depression, researchers proposed that depression is characterized by increased elaboration of negative information, difficulties in disengaging from negative material, and deficits in cognitive control when processing negative information (Gotlib & Joormann, 2010). In other words, impairment in emotional processing is a core pathological change in MDD. Consistently, our findings demonstrated that compared with SAD, GM volume decrease in the parahippocampal gyrus, and subsequent impairment of negative emotion processing is a potential pathological mechanism in MDD. Furthermore, the specific changes in neural structure can be a reliable marker for MDD.

### Limitations

There are some limitations to our study. (1) Although we performed a subgroup analysis, the methodological heterogeneity of the VBM studies may negatively impact our results. Therefore, well-designed VBM studies are needed to confirm our findings. (2) Due to the lack of studies on SAD, we have only conducted multiple comparison corrections for some results, which could affect the reliability of our results. As the incidence rate of SAD is increasing annually, it is necessary to conduct high-quality neuroimaging studies on SAD. (3) Voxel-based meta-analyses are based on summarized coordinates from published studies rather than raw data, which may result in less accurate results. (4) Publication bias may exist and affect the reliability of our results.

## Conclusions

Despite the aforementioned limitations, the current meta-analysis indicated that GM volume decrease in the right parahippocampal gyrus, especially in the amygdala, may be related to the high comorbidity rate and similar cognitive patterns in MDD and SAD. Besides, the current meta-analysis identified a unique pattern of GM decrease, with lower GM volume in the right lentiform nucleus and medial frontal gyrus in patients with SAD and lower GM volume in the left parahippocampal gyrus in patients with MDD. This pattern of GM volume decrease is consistent with the clinical manifestations of MDD and SAD. These findings offer a better understanding of the underlying neuropathogenesis of MDD and SAD and provide potential imaging markers for MDD and SAD.

### Supplementary Information

Below is the link to the electronic supplementary material.

Supplementary file1 (DOCX 28 KB)

Supplementary file2 (DOCX 35.9 KB)

## Data Availability

Not applicable.

## Abbreviations

MDD: Major depressive disorder
SAD: Social anxiety disorder
GM: Gray matter
VBM: Voxel-based morphometry
MRI: Magnetic resonance imaging
DSM: Diagnostic and Statistical Manual of Mental Disorders
HCs: Healthy controls
MOG: Middle occipital gyrus
SMA: Supplementary motor area
ALE: Activation likelihood estimation
ROI: Region of interest
PRISMA: Preferred Reporting Items for Systematic Reviews and Meta-analyses
SVC: Small volume correction
FDR: False discovery rate
NMDA: N-methyl-d-aspartate
