# Supplementary material for: Gray matter abnormalities in patients with major depressive disorder and social anxiety disorder: a voxel-based meta-analysis
Source: Brain Imaging Behav. 2023 Sep 19;17(6):749–63. doi: 10.1007/s11682-023-00797-z (PMC10733224; doi:10.1007/s11682-023-00797-z)
Supplement: Supplementary file 1 — Supplementary file1 (DOCX 28 KB) [file 11682_2023_797_MOESM1_ESM.docx]

| Supplemental Table 1. The search strategy used in the PubMed database.  (Search strategies were also used for other electronic databases.) | |
| --- | --- |
| NO. | Search items |
| #1 | Depressive Disorders, Major |
| #2 | Major Depressive Disorders |
| #3 | Major Depressive Disorder |
| #4 | Clinical depression, Major |
| #5 | Major clinical depression |
| #6 | Unipolar depression, Major |
| #7 | Major unipolar depression |
| #8 | Unipolar disorder, Major |
| #9 | Major unipolar disorder |
| #10 | Severe depression |
| #11 | Classic depression, Major |
| #12 | Major classic depression |
| #13 | #1 or #2-#12 |
| #14 | Phobias, Social |
| #15 | Social Phobia |
| Supplemental Table 1. continued. | |
| #16 | Social Phobias |
| #17 | Social Anxiety Disorder |
| #18 | Anxiety Disorder, Social |
| #19 | Anxiety Disorders, Social |
| #20 | Disorder, Social Anxiety |
| #21 | Disorders, Social Anxiety |
| #22 | Social Anxiety Disorders |
| #23 | #14 or #15-#22 |
| #24 | voxel-based morphometry |
| #25 | voxel based morphometry |
| #26 | VBM |
| #27 | #24 or #25-#26 |
| #28 | #13 or #16 and #27 |
