# Supplementary material for: Gray matter abnormalities in patients with major depressive disorder and social anxiety disorder: a voxel-based meta-analysis
Source: Brain Imaging Behav. 2023 Sep 19;17(6):749–63. doi: 10.1007/s11682-023-00797-z (PMC10733224; doi:10.1007/s11682-023-00797-z)
Supplement: Supplementary file 2 — Supplementary file2 (DOCX 35.9 KB) [file 11682_2023_797_MOESM2_ESM.docx]

Supplemental Table 2. The quality assessment of the included MRI studies.

| NO. | Included studies (First author, year) | MRI design described | Age reported | Sample gender reported | Matched control group | Sample handedness reported | Ethics approval reported | Image acquisition described | Image processing described | Statistical MRI-analysis described | Software package specified | Multiple comparison correction described | Figures and tables |
| --- | --- | --- | --- | --- | --- | --- | --- | --- | --- | --- | --- | --- | --- |
| **1. MDD n=34** | | | | | | | | | | | | | |
| 1 | Alemany et al., 2013 | Y | Y | Y | A, S, H | Y | Y | Y | Y | Y | Y | Y | Y |
| 2 | Arnone et al., 2013 | Y | Y | Y | A, S, H | Y | Y | Y | Y | Y | Y | Y | Y |
| 3 | Bergouignan et al., 2009 | Y | Y | Y | A, S, H | Y | Y | Y | Y | Y | Y | Y | Y |
| 4 | Chaney et al., 2014 | Y | Y | Y | A, S | Y | Y | Y | Y | Y | Y | Y | Y |
| 5 | Frodl et al., 2008 | Y | Y | Y | A, S, H | Y | Y | Y | Y | Y | Y | Y | Y |
| 6 | Grieve et al., 2013 | Y | Y | Y | A, S, E | Y | Y | Y | Y | Y | Y | Y | Y |
| 7 | Hwang et al., 2010 | Y | Y | Y | A, S, E | Y | Y | Y | Y | Y | Y | N | Y |
| 8 | Kim et al., 2008 | Y | Y | Y | A, S, H, E | Y | Y | Y | Y | Y | Y | Y | Y |
| 9 | Lai et al., 2015 | Y | Y | Y | A, S, H, E | Y | Y | Y | Y | Y | Y | Y | Y |
| 10 | Machino et al., 2014 | Y | Y | Y | A, S | Y | Y | Y | Y | Y | Y | Y | Y |
| 11 | Mak et al., 2009 | Y | Y | Y | A, S, E | Y | Y | Y | Y | Y | Y | N | Y |
| 12 | Peng et al., 2011 | Y | Y | Y | A, S, H, E | Y | Y | Y | Y | Y | Y | N | Y |
| 13 | de Azevedo-Marques Périco et al., 2011 | Y | Y | Y | A, S, H, E | Y | Y | Y | Y | Y | Y | Y | Y |
| Supplemental Table 2. continued. | | | | | | | | | | | | | |
| 14 | Scheuerecker et al., 2010 | Y | Y | Y | A, S, H | Y | Y | Y | Y | Y | Y | N | Y |
| 15 | Shah et al., 1998 | Y | Y | Y | A, S, E | Y | Y | Y | Y | Y | Y | N | Y |
| 16 | Stratmann et al., 2014 | Y | Y | Y | A, S, H | Y | Y | Y | Y | Y | Y | Y | Y |
| 17 | Tang et al., 2007 | Y | Y | Y | A, S, E | Y | Y | Y | Y | Y | Y | Y | Y |
| 18 | van Tol et al., 2010 | Y | Y | Y | A, S, H, E | Y | Y | Y | Y | Y | Y | Y | Y |
| 19 | Vasic et al., 2008 | Y | Y | Y | A, S, H, E | Y | Y | Y | Y | Y | Y | N | Y |
| 20 | Wagner et al., 2011 | Y | Y | Y | A, S, H, E | Y | Y | Y | Y | Y | Y | Y | Y |
| 21 | Zhang et al., 2012 | Y | Y | Y | A, S, H, E | Y | Y | Y | Y | Y | Y | Y | Y |
| 22 | Zou et al., 2010 | Y | Y | Y | A, S, H, E | Y | Y | Y | Y | Y | Y | Y | Y |
| 23 | Straub et al., 2019 | Y | Y | Y | A, S, H, E | Y | Y | Y | Y | Y | Y | Y | Y |
| 24 | Wang et al., 2019 | Y | Y | Y | A, S, H | Y | Y | Y | Y | Y | Y | Y | Y |
| 25 | Li et al., 2019 | Y | Y | Y | A, S, H | Y | Y | Y | Y | Y | Y | Y | Y |
| 26 | Sun et al., 2020 | Y | Y | Y | A, S, H, E | Y | Y | Y | Y | Y | Y | Y | Y |
| 27 | Chen et al., 2020 | Y | Y | Y | A, S, H | Y | Y | Y | Y | Y | Y | Y | Y |
| 28 | Zhang et al., 2020 (1) | Y | Y | Y | A, S, H, E | Y | Y | Y | Y | Y | Y | Y | Y |
| 29 | Xu et al., 2019 | Y | Y | Y | A, S, E | Y | Y | Y | Y | Y | Y | Y | Y |
| 30 | Meng et al., 2020 | Y | Y | Y | A, S, H, E | Y | Y | Y | Y | Y | Y | Y | Y |
| 31 | Lu et al., 2019 | Y | Y | Y | A, S, H, E | Y | Y | Y | Y | Y | Y | Y | Y |
| 32 | Ma et al., 2021 | Y | Y | Y | A, S, H, E | Y | Y | Y | Y | Y | Y | Y | Y |
| 33 | Burhanoglu et al., 2021 | Y | Y | Y | A, S, E | Y | Y | Y | Y | Y | Y | Y | Y |
| Supplemental Table 2. continued. | | | | | | | | | | | | | |
| 34 | Zhang et al., 2020 (2) | Y | Y | Y | A, S, H, E | Y | Y | Y | Y | Y | Y | Y | Y |
| **2. SAD n=10** | | | | | | | | | | | | | |
| 1 | Talati et al., 2013 | Y | Y | Y | A, S, E | N | Y | Y | Y | Y | Y | Y | Y |
| 2 | Bas-Hoogendam et al., 2017 | Y | Y | Y | A, S, H, E | Y | Y | Y | Y | Y | Y | Y | Y |
| 3 | Irle et al., 2014 | Y | Y | Y | A, S, H, E | Y | Y | Y | Y | Y | Y | N | Y |
| 4 | Kawaguchi et al., 2016 | Y | Y | Y | A, S | N | Y | Y | Y | Y | Y | Y | Y |
| 5 | Meng et al., 2013 | Y | Y | Y | A, S, E | N | Y | Y | Y | Y | Y | Y | Y |
| 6 | Tükel et al., 2015 | Y | Y | Y | A, S, H, E | Y | Y | Y | Y | Y | Y | Y | Y |
| 7 | Zhao et al., 2017 | Y | Y | Y | A, S, H, E | Y | Y | Y | Y | Y | Y | Y | Y |
| 8 | Liu et al., 2021 | Y | Y | Y | A, S, E | N | Y | Y | Y | Y | Y | Y | Y |
| 9 | Mansson et al., 2016 | Y | Y | Y | A, S, H, E | Y | Y | Y | Y | Y | Y | Y | Y |
| 10 | Zhang et al., 2022 | Y | Y | Y | A, S, H, | Y | Y | Y | Y | Y | Y | Y | Y |
| Note: Y, yes; N, no; U, unclear; A, age; S, sex; H, handedness; E, education. | | | | | | | | | | | | | |
